# LncRNA SNHG6 enhances the radioresistance and promotes the growth of cervical cancer cells by sponging miR-485-3p

**DOI:** 10.1186/s12935-020-01448-9

**Published:** 2020-08-31

**Authors:** Jin Liu, Xiaojiao Liu, Rong Li

**Affiliations:** 1 Department of Obstetrics and Gynecology, The First Affiliated Hospital of Xi’an Jiaotong University, No. 277 Yanta West Road, Xi’an, 710061 Shaanxi China; 2 Department of Mammary Gland & Thyroid Surgery, Sichuan Gem Flower Hospital, No. 26 Tongjixiang Road, Chengdu, 610213 Sichuan China

**Keywords:** SNHG6, miR-485-3p, STYX, Cervical cancer

## Abstract

**Background:**

Cervical cancer (CC) is the one of most common malignant gynecological tumors, which is characterized with the high mortality and recurrence rate. Previous studies have elucidated the oncogenic role of small nucleolar RNA host gene 6 (SNHG6) in some types of human cancers, whereas it is unclear whether it functions as an oncogene in CC. This study was aimed at unveiling the role of SNHG6 in CC.

**Methods:**

qRT-PCR analysis was implemented to evaluate the expression levels of SNHG6, miR-485-3p and STYX in CC cells. RNA pull down assay and luciferase reporter assay were conducted to verify the interaction between miR-485-3p and SNHG6 or STYX. Functional assays, such as colony formation assay, JC-1 assay and TUNEL assay were applied to detect the biological behaviors of CC cells. The resistance of CC cells to radiation was evaluated by colony formation assay.

**Results:**

SNHG6 was expressed at a high level in CC cells. Silenced SNHG6 suppressed cell proliferation but promoted cell apoptosis. Additionally, silenced SNHG6 could sensitize CC cells to radiation treatment. miR-485-3p could bind to both SNHG6 and STYX. Knockdown of miR-485-3p or overexpression of STYX could abolish the effects of SNHG6 silencing on CC cell growth.

**Conclusions:**

LncRNA SNHG6 enhances the radioresistance of CC cells and promotes CC cell growth by sponging miR-485-3p to release STYX.

## Background

Cervical cancer (CC) is a malignant cancer worldwide and endangers the health of females [1–3]. Studies have shown that no less than 530,000 people are diagnosed with CC each year and 280,000 people are passed away because of this cancer [1–3]. Even if the development in surgery and medicine therapy, the overall survival of CC patients remains pessimistic [4, 5]. Therefore, the molecular mechanism underlying CC progression still need to be further identified. The aim of our present study was to explore the mechanism underneath the progression of CC.

Long non-coding RNAs (lncRNAs) are transcripts almost without the ability to code proteins, which are characterized with the length more than 200 nt. Studies have revealed the important role of lncRNAs in regulating protein-coding genes and carcinogenesis [6]. LncRNAs can modulate their target genes at transcriptional or post-transcriptional level. Functionally, lncRNAs can affect various biological processes in cancers, such as cell proliferation, migration and invasion [7, 8]. For instance, lncRNA PVT1 can reduce the radioresistance of non-small cell lung cancer cells by regulating the expression of miR-195 [9]. LncRNA EWSAT1 can activate cell proliferation by regulating miR-326 and miR-330-5p in nasopharyngeal carcinoma [10]. LncRNA ncRuPAR can inhibit tumor growth and metastasis in gastric cancer by regulating PAR-1 [11]. Small nucleolar RNA host genes (SNHG family) have been widely reported as the modulators in tumorigenesis and cancer progression [12–15]. Among which, small nucleolar RNA host gene 6 (SNHG6) has been identified as an oncogene in gastric cancer, hepatocellular carcinoma, colorectal cancer, glioma and lung adenocarcinoma [16–20]. However, the function of SNHG6 in CC remains to be explored. Thence, this study focused on the function and mechanism of SNHG6 in CC.

## Materials and methods

### Tissue samples

CC tissues and adjacent normal tissues (n = 56 each group) used in this study were collected from The First Affiliated Hospital of Xi’an Jiaotong University between Jan, 2019 and Feb, 2020. Patients enrolled in this study didn’t receive any kind of adjuvant therapies before surgery. All patients had signed the written inform consent before surgery. The Ethics Committee of the First Affiliated Hospital of Xi’an Jiaotong University had approved this study. All samples were immediately preserved in the liquid nitrogen and stored at − 80 °C as soon as they were collected from patients.

### Cell lines

Normal human cervical epithelial cell line (HCerEpiC) and CC cell lines (C-33 A, SiHa, HeLa, CaSki, HT-3) were purchased from Chinese Academy of Sciences (Shanghai, China). All cell lines were cultured in DMEM (Invitrogen, Carlsbad, CA) supplemented with 10% fetal bovine serum (FBS) and 1% antibiotics. Cell lines were placed in a humidified incubator under a condition of 5% CO_2_ and 37 °C.

### Quantitative real-time polymerase chain reaction (qRT-PCR)

Total RNA extraction was accomplished by the use of Trizol reagent (Thermo Fisher Scientific, Waltham, MA), then reversely transcribed into cDNA with PrimeScript™ RT reagent kit as instructed (Takara, Shiga, Japan). TaqMan™ MicroRNA Reverse Transcription Kit was utilized for the reverse transcription of miRNAs. TB Green^®^ Premix Ex Taq™ (Takara, Tokyo, Japan) was used for qRT-PCR analysis. The fold-change of gene expression was evaluated by 2^−ΔΔCt^ method. GAPDH gene or U6 was used as the internal controls.

### Transfection

To stably silence SNHG6 and STYX, the designed shRNAs and negative control shRNAs (sh-NC) were procured from GenePharma (Shanghai, China) and transfected into SiHa and HeLa cells by using Lipofectamine 3000 (Invitrogen). To overexpress STYX, the whole sequence of it was cloned into pcDNA3.1 vector so as to generate pcDNA3.1/STYX vector. The empty vector was used as the negative control (pcDNA3.1-NC). miR-485-3p mimics/inhibitor and NC mimics/inhibitor were designed by RiboBio (Guangzhou, China). Cells were harvested at 48 h’ post-transfection. This experiment was repeated at least three times.

### Colony formation assay

SiHa and HeLa cells were collected and seeded into 6-well plate with 500 cells per well followed by the incubation for 14 days. Colonies were fixed with 4% paraformaldehyde and stained by 0.1% crystal violet. The number of colonies was counted manually. This experiment was repeated at least three times.

### EdU assay

SiHa and HeLa cells were cultured in 96-well plate (1 × 10^4^ cells/well) in presence of EdU medium diluent. After fixation, proliferative cells were determined by using the EdU assay Kit (Ribobio). Cell nuclei were stained with DAPI and observed under a fluorescent microscope (Olympus, Tokyo, Japan). This experiment was repeated at least three times.

### JC-1 assay

Cells cultured in 96-well plate were incubated overnight, then centrifuged and treated with JC-1 dye. Thirty minutes after incubation, the change of mitochondrial transmembrane potential (ΔΨm) was examined by a fluorescent plate reader and imaged using a fluorescence microscope. This experiment was repeated at least three times.

### TUNEL assay

Cells (1 × 10^4^) on culture slides were fixed with 4% paraformaldehyde and washed in PBS, then treated with 0.1% TritonX-100 for re-suspension. Apoptotic cells were examined by applying 50 μl TUNEL Detection Kit in line with direction (Roche, Basel, Switzerland) and incubated at 37 °C for 60 min. Cell nuclei were dyed with DAPI. After washing with PBS for three times, observed the samples under a microscope. This experiment was repeated at least three times.

### Survival fraction assay

Transfected cells were plated into 6-well with 500 cells in each well, and then treated with ionizing radiation at 0, 2, 4, 6, 8 and 10 Gy. After incubated from 14 days, cells were fixed and stained with crystal violet. Finally, the survival colonies were counted. This experiment was repeated at least three times.

### Fish

Cells (6 × 10^4^) were fixed with 4% paraformaldehyde for 15 min, then dehydrated and air-dried for hybridization with the specific SNHG6-FISH probe (Ribobio, Guangzhou, China) according to the protocol of FISH kit (Ribobio). After stained cell nuclei with DAPI dye, samples were finally photographed and observed under a microscope. This experiment was repeated at least three times.

### Subcellular fraction

SiHa and HeLa cells (1 × 10^6^ cells/well) were subjected to PARIS™ Kit (Invitrogen) after washing in precooled PBS. Afterwards, the cell fractionation buffer and cell disruption buffer were used to isolate cytoplasm and nucleus. U6 and GAPDH were separately used as the internal controls for the nuclear or cytoplasmic RNA. Cytoplasmic or nuclear content of SNHG6 was monitored by qRT-PCR. This experiment was repeated at least three times.

### RNA immunoprecipitation (RIP)

MS2-RIP assay were accomplished in SiHa and HeLa cells using GFP antibody and RIP RNA-Binding Protein Immunoprecipitation Kit as per direction (Millipore, Bedford, MA). Cells were co-transfected with pMS2-GFP and MS2-SNHG6 or MS2 constructs for 48 h. The final precipitates were assayed by qRT-PCR. This experiment was repeated at least three times.

### Luciferase reporter assay

The SNHG6 or STYX fragments covering the miR-485-3p target sites including wild-type and mutant type were cloned into pmirGLO vector (Promega, Madison, WI, USA), termed SNHG6-WT/Mut and STYX-WT/Mut vectors. The vectors were co-transfected with miR-485-3p mimics or NC-mimics into SiHa and HeLa cells for 48 h, and the luciferase activity was measured using Luciferase Reporter Assay System (Promega) with the Renilla luciferase as the internal control. This experiment was repeated at least three times.

### RNA pull down assay

miR-485-3p sequences covering with or without the complementary base pairing to SNHG6 or STYX target sequences (wild-type and mutant) were designed and biotinylated to Bio-miR-485-3p-WT/Mut probes. The lysates isolated from 1 × 10^6^ cells were mixed with the Bio-NC, Bio-miR-485-3p-WT/Mut for 1 h, then incubated with the streptavidin beads for 30 min. After washing, RNAs in the complexes were purified with TRIzol reagent (Thermo Fisher Scientific, Waltham, MA, USA). The recovered RNAs were quantified by qRT-PCR. This experiment was repeated at least three times.

### Statistical analysis

All data were obtained from three or more independent experiments and expressed as the mean ± standard deviation (SD). Data were processed by PRISM 6 (GraphPad, San Diego, CA). Differences between two groups were analyzed using Student’s *t* test, while the differences among more than two groups were analyzed by one-way or two-way ANOVA. Data were statistically significant when the p (possibility) value less than 0.05.

## Results

### SNHG6 promotes CC cell growth and enhances the radioresistance

**Fig. 1:**
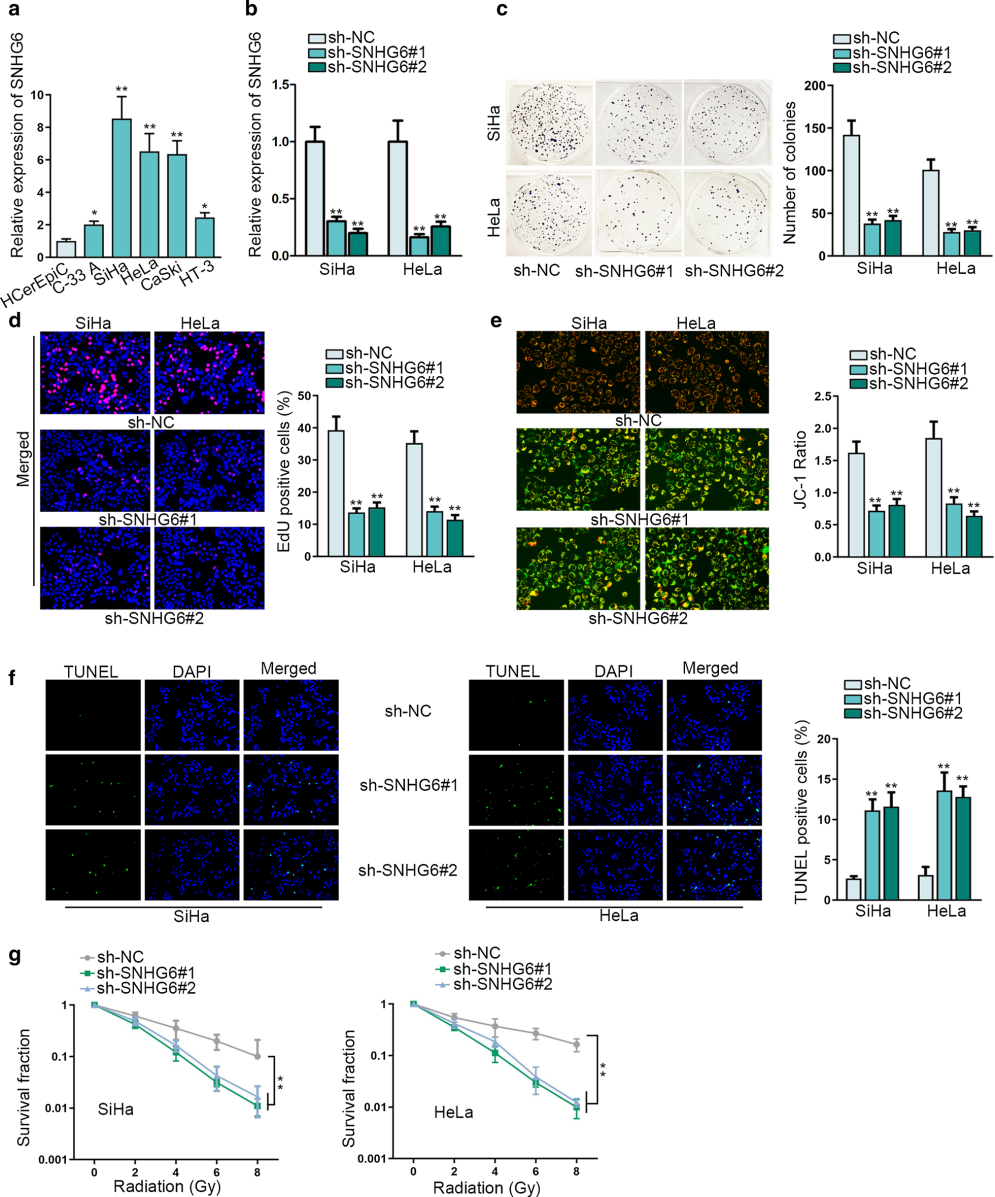
SNHG6 promotes CC cell growth and enhances the radioresistance. **a** Expression of SNHG6 was probed in normal human cervical epithelial cell line (HCerEpiC) and CC cell lines (C-33 A, SiHa, HeLa, CaSki and HT-3) via qRT-PCR. **b** SNHG6 expression was inhibited by the specific shRNAs (sh-SNHG6#1 and sh-SNHG6#2). Non-targeted shRNA was used as the negative control (sh-NC). Transfection efficiency was evaluated after 48 h by qRT-PCR. **c**, **d** Cell proliferation was identified via colony formation assay and EdU assay after two cells were transfected with SNHG6-specific shRNAs or control shRNA. **e**, **f** JC-1 assay and TUNEL assay tested the apoptosis in the early phase or later phase after silencing of SNHG6 with specific shRNAs. **g** The sensitivity of SiHa and HeLa cells to radiation was evaluated after knockdown of SNHG6 by colony formation assay. *p < 0.05, **p < 0.01

At first, we determined that SNHG6 was expressed at a high level in CC tissues compared with the adjacent normal tissues (Additional file 1: Figure S1A, **p < 0.01). Consistently, we obtained the results that SNHG6 was expressed higher in five CC cells (C-33 A, SiHa, HeLa, CaSki and HT-3), especially in SiHa, HeLa and CaSki cells, than that in the normal cervical epithelial cell (HCerEpiC) (Fig. 1a, *p < 0.05, **p < 0.01). Then, we designed loss-of function assays and transfected SNHG6-specific shRNAs (sh-SNHG6#1 and sh-SNHG6#2) into SiHa, HeLa and CaSki cells (Fig. 1b and Additional file 1: Figure S1B, **p < 0.01). Functionally, the number of colonies and the percentage of EdU-positive cells in three CC cells were decreased more than 50% after silencing of SNHG6 (Fig. 1c, d and Additional file 1: Figure S1C, D, **p < 0.01). Meanwhile, apoptosis condition was assessed in SNHG6-downregulated cells. As shown in Fig. 1e, f and Additional file 1: Figure S1E, F (**p < 0.01), the apoptosis rate was increased more than two times after knockdown of SNHG6. Finally, silenced SNHG6 enhanced the sensitivity of CC cells to radiation treatment (Fig. 1g and Additional file 1: Figure S1G, **p < 0.01). In a word, SNHG6 can exert oncogenic functions in CC by facilitating cell growth and enhancing radioresistance.

### miR-485-3p can bind with SNHG6 in CC cells

**Fig. 2:**
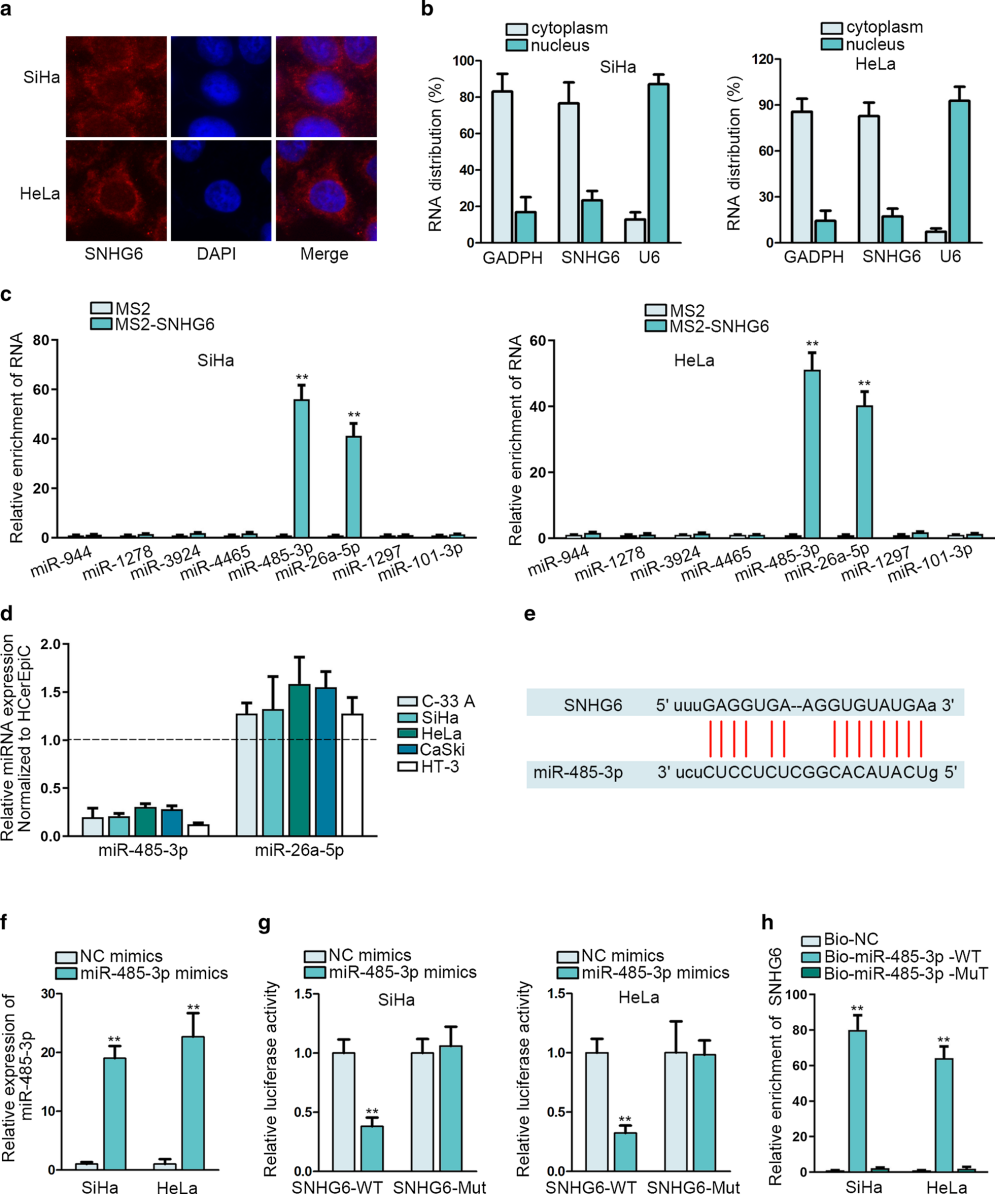
miR-485-3p can bind with SNHG6 in CC cells. **a**, **b** Subcellular fraction assay and FISH assay searched the SNHG6 location in SiHa and HeLa cells. Blue (DAPI): nuclei, Red: SNHG6. **c** MS2-RIP assay searched the binding ability of miRNAs to SNHG6. **d** Detection of miR-485-3p expression and miR-26a-5p expression in CC cells via qRT-PCR. **e** Binding sites of SNHG6 to miR-485-3p obtained from ENCORI were presented. **f** Overexpression efficiency of miR-485-3p was assessed via qRT-PCR in SiHa and HeLa cells after miR-485-3p mimics or NC mimics were transfected into above cells at 48 h. **g**, **h** Luciferase reporter assay and RNA pull down assay proved the binding of SNHG6 to miR-485-3p in SiHa and HeLa cells. **p < 0.01

The underlying molecular mechanism of SNHG6 was explored through mechanism investigation. At first, we identified the cytoplasmic localization of SNHG6 in CC cells via subcellular fraction assay and FISH assay (Fig. 2a, b). Next, we explored whether cytoplasmic SNHG6 could interact with miRNA to post-transcriptionally modulate mRNAs. ENCORI website (http://starbase.sysu.edu.cn/index.php) was used to search the miRNAs that possess the binding sites with SNHG6. Meanwhile, we applied MS2-RIP assay to prove the interaction of SNHG6 with predicted miRNAs. The highest enrichment was found in miR-485-3p and miR-26a-5p (Fig. 2c, **p < 0.01). Expression of miR-485-3p and miR-26a-5p were detected in CC cells via qRT-PCR analysis. As a result, we found that miR-485-3p was expressed lowly in CC cells (Fig. 2d). The binding sites of SNHG6 in miR-485-3p seed region were obtained from ENCORI and shown in Fig. 2e. The efficiency of miR-485-3p overexpression in SiHa and HeLa cells was assessed via qRT-PCR (Fig. 2f, **p < 0.01). Next, luciferase reporter assay and RNA pull down assay further proved the binding of SNHG6 to miR-485-3p (Fig. 2g, h, **p < 0.01). In brief, miR-485-3p can bind to SNHG6 in CC cells.

### STYX is the downstream target of miR-485-3p and functions as an oncogene in CC cells

**Fig. 3:**
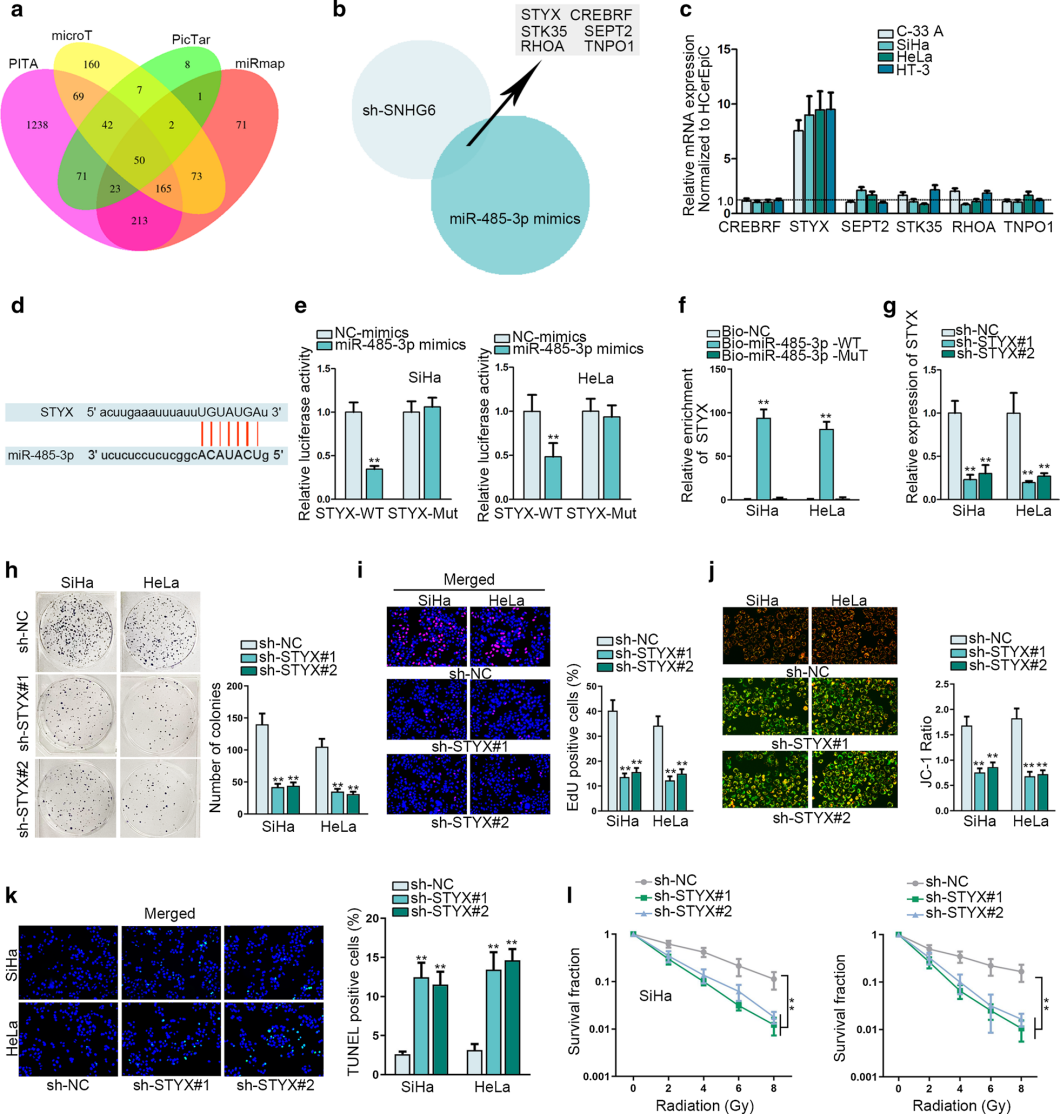
STYX is the downstream target of miR-485-3p and functions as an oncogene in CC cells. **a** Target genes of miR-485-3p were explored via ENCORI website in accordance with the intersection of four bioinformatics tools. **b** qRT-PCR analysis of mRNAs that could be affected by silenced SNHG6 or overexpressed miR-485-3p. **c** Expression levels of six candidate mRNAs (CREBRF, STYX, SEPT2, STK35, RHOA and TNPO1) were assessed via qRT-PCR in CC cells. **d** Binding site of STYX to miR-485-3p obtained from ENCORI was presented. **e** Luciferase reporter assay revealed the luciferase activity of STYX-WT or STYX-Mut in cells co-transfected with NC mimics or miR-485-3p mimics. **f** RNA pull down assay detected the enrichment of STYX in cells incubated with bio-NC, bio-miR-485-3p-WT or bio-miR-485-3p-Mut. **g** Inhibition efficiency of STYX was tested via qRT-PCR in SiHa and HeLa cells transfected with sh-NC or shRNA targeted to STYX. **h**, **i** Cell proliferation was identified via colony formation assay and EdU assay. **j**, **k** JC-1 assay and TUNEL assay assessed the apoptosis rate of SiHa and HeLa cells after silencing with STYX. **l** The sensitivity of CC cells to radiation was measured after silencing of STYX. **p < 0.01

The potential targets of miR-485-3p were predicted and searched out. Searching from the online database ENCORI, 50 mRNAs of miR-485-3p were searched out with the intersection of PITA, microT, picTar and miRmap (Fig. 3a). Next, these 50 mRNAs were subjected to qRT-PCR six of them (CREBRF, STYX, SEPT2, STK35, RHOA and TNPO1) were uncovered to be significantly downregulated in response to SNHG6 silencing or miR-485-3p overexpression (Fig. 3b). Subsequently, their expression was assessed via qRT-PCR in CC cells and only STYX was highly expressed in CC cells (Fig. 3c). Meanwhile, the binding sites of STYX to miR-485-3p were predicted and provided (Fig. 3d). Thence, we assured the binding of STYX to miR-485-3p via luciferase reporter assay and RNA pull down assay (Fig. 3e, f, **p < 0.01). The inhibitory efficiency of STYX expression was tested via qRT-PCR in SiHa and HeLa cells (Fig. 3g, **p < 0.01). Furthermore, STYX depletion led to the reduction of cell proliferation (Fig. 3h, i, **p < 0.01). In addition, we identified the accelerative impact of STYX knockdown on CC cell apoptosis (Fig. 3j, k, **p < 0.01). The sensitivity of CC cells to radiation was also strengthened by the silencing of STYX (Fig. 3l, **p < 0.01). In a word, STYX can function as an oncogene and bind with miR-485-3p in CC cells.

### SNHG6/miR-485-3p/STYX axis can modulate CC cell growth

**Fig. 4:**
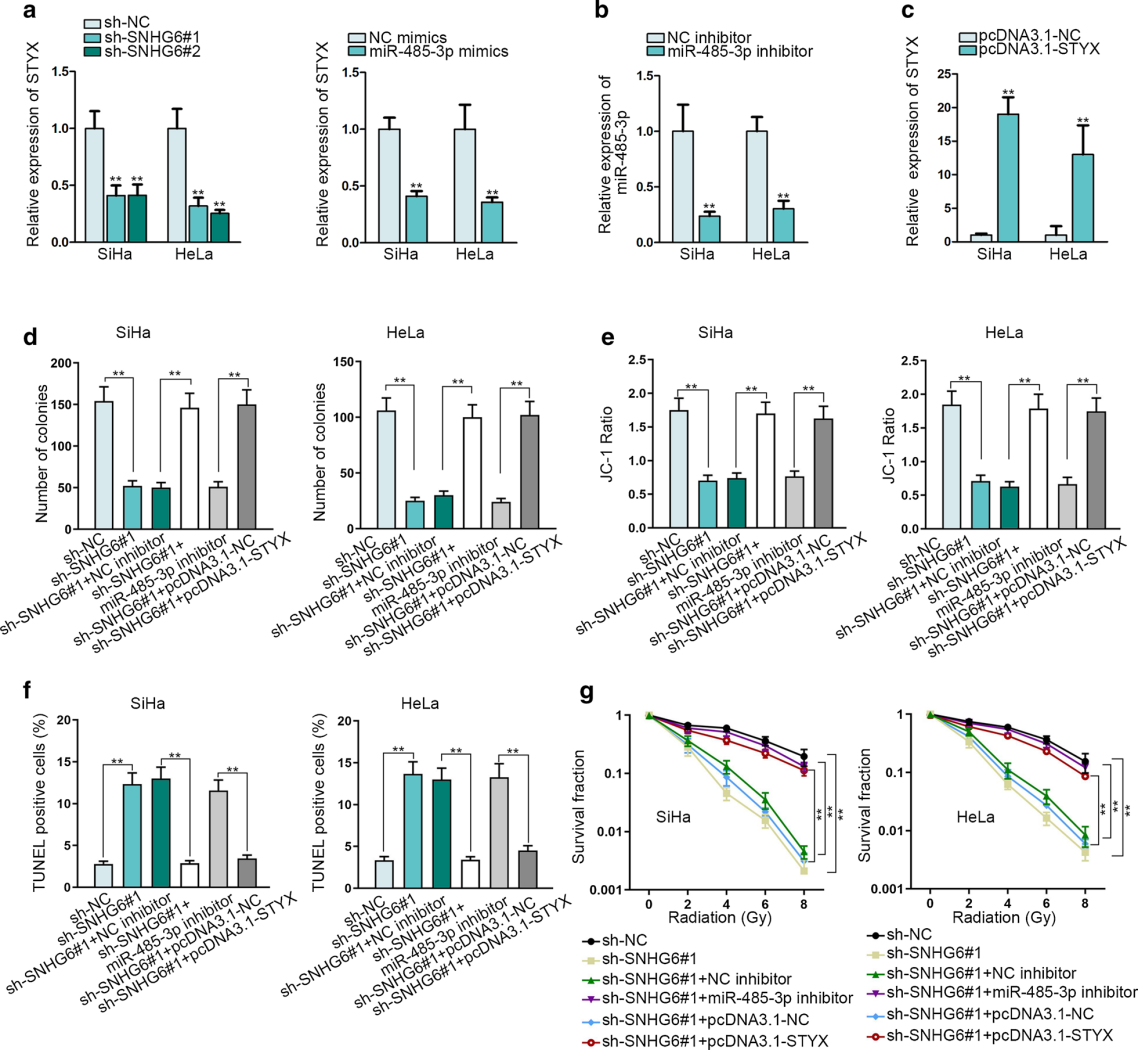
SNHG6/miR-485-3p/STYX axis modulates CC cell growth. **a** The expression of STYX mRNA was identified via qRT-PCR when SNHG6 was silenced or miR-485-3p was overexpressed. **b**, **c** Inhibition efficiency of miR-485-3p and overexpression efficiency of STYX were assessed via qRT-PCR in SiHa and HeLa cells. **d** Colony formation assay probed the proliferation of SiHa and HeLa cells. Cells were transfected with sh-NC, sh-SNHG6#1, sh-SNHG6#1 + NC inhibitor, sh-SNHG6#1 + miR-485-3p inhibitor, sh-SNHG6#1 + pcDNA3.1-NC, sh-SNHG6#1 + pcDN3.1/STYX, respectively. **e**, **f** JC-1 assay and TUNEL assay tested the cell apoptosis of SiHa and HeLa cells. Cells were transfected with sh-NC, sh-SNHG6#1, sh-SNHG6#1 + NC inhibitor, sh-SNHG6#1 + miR-485-3p inhibitor, sh-SNHG6#1 + pcDNA3.1-NC, sh-SNHG6#1 + pcDN3.1/STYX, respectively. **g** Ionizing radiation investigated the cell radioresistance. Cells were transfected with sh-NC, sh-SNHG6#1, sh-SNHG6#1 + NC inhibitor, sh-SNHG6#1 + miR-485-3p inhibitor, sh-SNHG6#1 + pcDNA3.1-NC, sh-SNHG6#1 + pcDN3.1/STYX, respectively. *p < 0.05, **p < 0.01

Finally, the involvement of miR-485-3p or STYX in SNHG6-mediated cellular processes was probed. The level of STYX mRNA was found to be reduced by the downregulation of SNHG6 or the overexpression of miR-485-3p (Fig. 4a, **p < 0.01). Before the rescue assays, miR-485-3p and STYX were separately silenced or overexpressed in two CC cells (Fig. 4b, c, **p < 0.01). The proliferation suppressed by SHNG6 silencing was recovered after co-transfection with miR-485-3p inhibitor or STYX expression vector (Fig. 4d and Additional file 2: Figure S2A, **p < 0.01). Simultaneously, the apoptosis rate enhanced by the downregulation of SNHG6 was decreased again by the inhibition of miR-485-3p or the ectopic expression of STYX (Fig. 4e, f and Additional file 2: Figure S2B, C, **p < 0.01). Finally, the sensitivity of CC cells to radiation induced by SNHG6 knockdown was reversed again after miR-485-3p depletion or STYX overexpression (Fig. 4g, **p < 0.01). To summarize, SNHG6/miR-485-3p/STYX axis exerts oncogenic functions in CC.

## Discussion

Previous studies have elucidated that lncRNAs are essential regulators and tumor facilitators in CC. For example, highly expressed lncRNA DANCR in CC cells can promote cell growth and invasion via regulating miR-335-5p and ROCK1 [21]; LncRNA NEAT1 can modulate PI3K/AKT signaling to affect CC progression [22]; LncRNA XLOC_006390 can facilitate tumor growth and metastasis in cervical cancer through modulating SET8 expression [23]. In the present study, we found that SNHG6 level was aberrantly elevated in CC cells via qRT-PCR analysis. Meanwhile, functional assays were also implemented to assess the functions of SNHG6 in CC cells. It was found that cell growth was markedly suppressed in response to the silencing of SNHG6. However, SNHG6 silencing accelerated CC cell apoptosis. Therefore, we confirmed that SNHG6 can function as an oncogene in CC.

microRNAs (miRNAs) are known as the molecules which can interact with lncRNAs. Recent years, the anti-oncogenic role of miRNAs has been elucidated in CC. For instance, miR-338-3p can reduce CC cell growth via regulating MACC1 and MAPK expression [24]. miR-489 is expressed lowly in CC and reduces the proliferation of CC cells [25]. miR-221-3p can regulate EMT and metastasis through binding to THBS2 in CC [26]. miR-485-3p has been reported as a tumor suppressor in breast cancer and glioblastoma [27–29]. In our study, we found that miR-485-3p could bind to SNHG6. Meanwhile, miR-485-3p was lowly expressed in CC cells. Functionally, upregulation of miR-485-3p led to the suppression of CC cell growth. Meanwhile, mechanism investigation unmasked that SNHG6 acted as a sponge for miR-485-3p. Thus, we identified the tumor-suppressive role of miR-485-3p and its interaction with SNHG6 in CC.

Precious studies have found that STYX can act as an oncogene in colorectal cancer [30]. Here, STYX was explored as the downstream target of miR-485-3p in CC. Moreover, functional assays prompted us to conclude that STYX acts as an oncogene in CC. Collectively, this study revealed that SNHG6/miR-485-3p/STYX axis contributes to the growth and radioresistance of CC cells, thus promoting CC progression. This study revealed a novel competing endogenous RNA (ceRNA) pathway in CC, which may contribute to uncovering novel therapeutic targets for CC.

## Conclusion

SNHG6 is firstly identified as the tumor promoter in CC. SNHG6 can function as a ceRNA to modulate miR-485-3p/STYX axis and further regulate CC progression.

## Supplementary information

**Additional file 1: Figure S1.** A. SNHG6 expression was evaluated in paired tissues obtained from 56 CC patients. B. SNHG6 silencing in CaSki cells with shRNAs. qRT-PCR analysis of the results after 48 h. C, D. Colony formation and EdU assay revealed the proliferative ability of CaSki cells. EdU: red, DAPI: blue. E-F. JC-1 and TUNEL assays determined the apoptosis rate of CaSki cells in the early phase or late phase after transfection with sh-SNHG6#1/#2. G. Radioresistance of CaSki cells was identified with colony formation assay after sh-SNHG6#1/2 transfection. **p < 0.01.

**Additional file 2: Figure S2.** A–C. Original images for the rescue assays shown in Fig. 4d–f.

## Data Availability

Not applicable.

## Abbreviations

SNHG6: Small nucleolar RNA host gene 6
lncRNAs: Long noncoding RNAs
miRNAs: microRNAs
mRNA: Messenger RNA
ceRNA: Competing endogenous RNA
WT: Wild-type
Mut: Mutant
EdU: 5-Ethynyl-20-deoxyuridine
TUNEL: The terminal deoxynucleotidyl transferase dUTP nick-end labelling
FISH: Fluorescent in situ hybridization
qRT-PCR: Quantitative real-time PCR
FBS: Fetal bovine serum
RNA immunoprecipitation: RIP
DMEM: Dulbecco’s Modified Eagle’s Medium
GAPDH: Glyceraldehyde-3-phosphate dehydrogenase
SD: Standard deviation
ANOVA: Analysis of variance
