# Supplementary figures and images for: LncRNA SNHG6 enhances the radioresistance and promotes the growth of cervical cancer cells by sponging miR-485-3p
Source: Cancer Cell Int. 2020 Aug 31;20:424. doi: 10.1186/s12935-020-01448-9 (PMC7457785; doi:10.1186/s12935-020-01448-9)

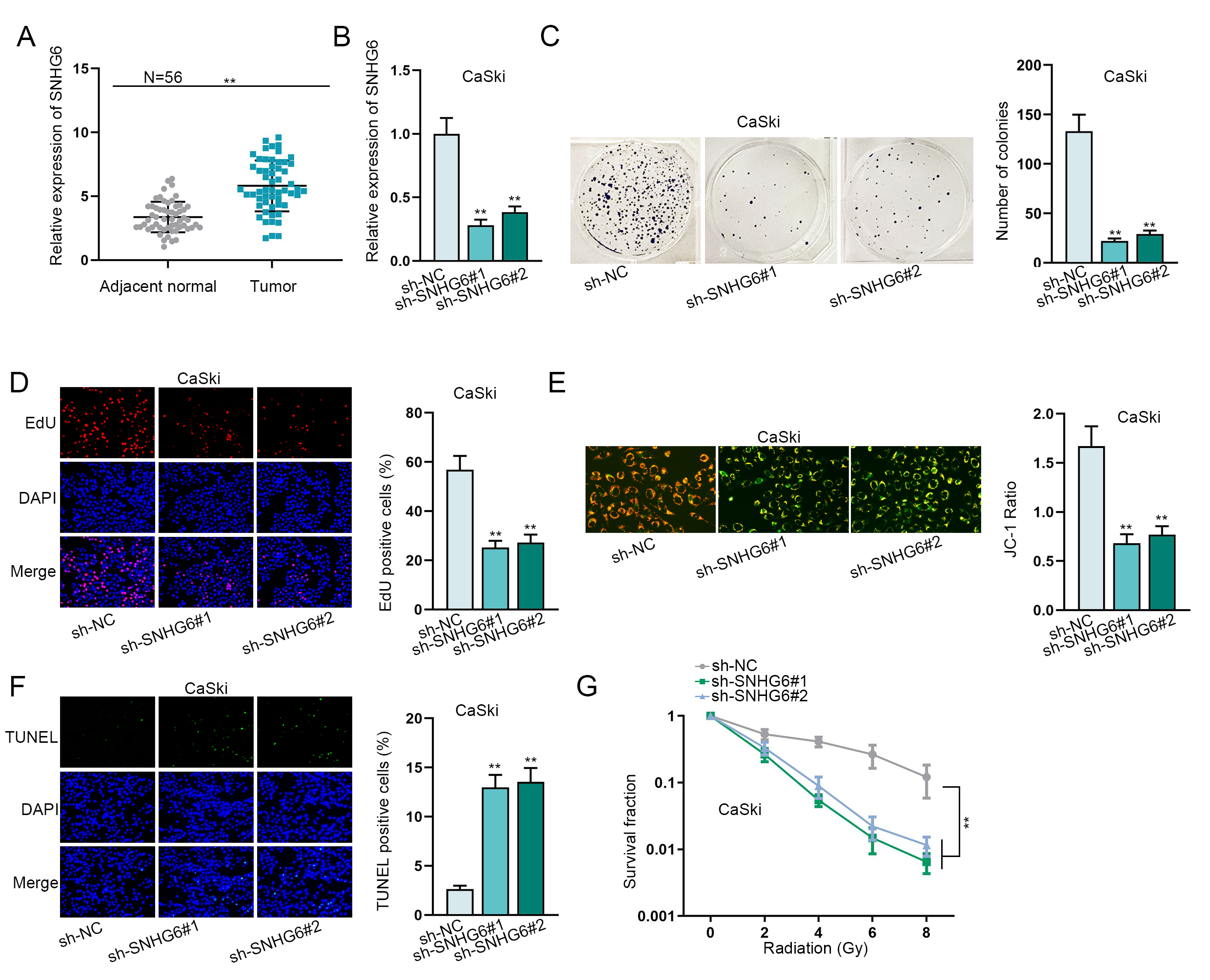

Supplement: Supplementary file 1 — Additional file 1: Figure S1. A. SNHG6 expression was evaluated in paired tissues obtained from 56 CC patients. B. SNHG6 silencing in CaSki cells with shRNAs. qRT-PCR analysis of the results after 48 h. C, D. Colony formation and EdU assay revealed the proliferative ability of CaSki cells. EdU: red, DAPI: blue. E-F. JC-1 and TUNEL assays determined the apoptosis rate of CaSki cells in the early phase or late phase after transfection with sh-SNHG6#1/#2. G. Radioresistance of CaSki cells was identified with colony formation assay after sh-SNHG6#1/2 transfection. **p < 0.01. [file 12935_2020_1448_MOESM1_ESM.tif]

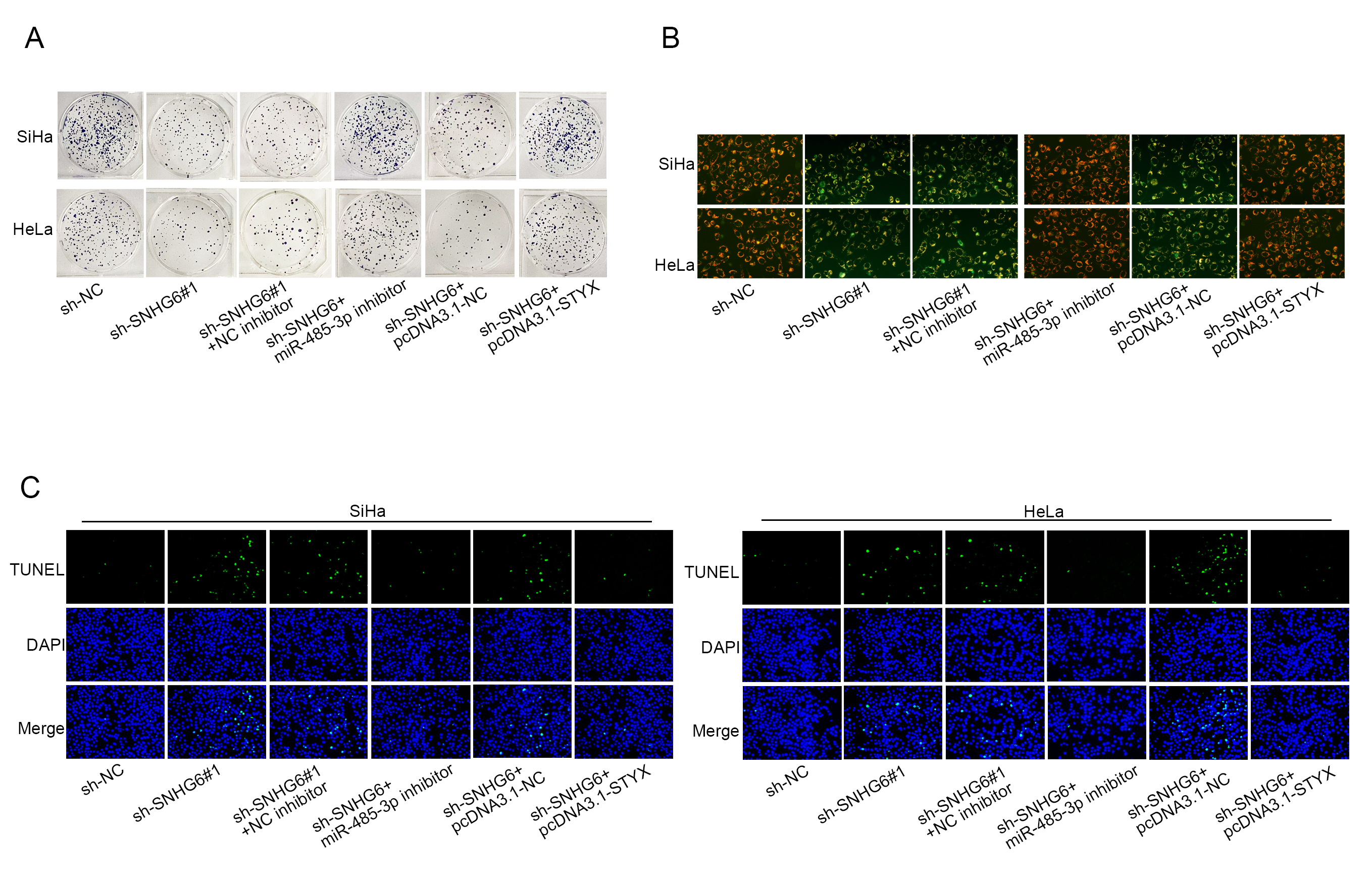

Supplement: Supplementary file 2 — Additional file 2: Figure S2. A–C. Original images for the rescue assays shown in Fig. 4d–f. [file 12935_2020_1448_MOESM2_ESM.tif]
